# The impact of impaired DNA mobility on gene electrotransfer efficiency: analysis in 3D model

**DOI:** 10.1186/s12938-021-00922-3

**Published:** 2021-08-21

**Authors:** Saša Haberl Meglič, Mojca Pavlin

**Affiliations:** 1 Faculty of Electrical Engineering, Laboratory of Biocybernetics, University of Ljubljana, Tržaška 25, 1000 Ljubljana, Slovenia; 2 Faculty of Medicine, Institute of Biophysics, University of Ljubljana, Vrazov trg 2, 1000 Ljubljana, Slovenia; 3 Faculty of Electrical Engineering, Group for Nano and Biotechnological Applications, University of Ljubljana, Tržaška 25, 1000 Ljubljana, Slovenia

**Keywords:** 3D in vitro model, Collagen gel, Diffusion, Electrophoresis, Gene electrotransfer, GFP, High-voltage pulse, Low-voltage pulse, Different polarity pulses

## Abstract

**Background:**

Gene electrotransfer is an established method that enables transfer of DNA into cells with electric pulses. Several studies analyzed and optimized different parameters of gene electrotransfer, however, one of main obstacles toward efficient electrotransfection in vivo is relatively poor DNA mobility in tissues. Our aim was to analyze the effect of impaired mobility on gene electrotransfer efficiency experimentally and theoretically. We applied electric pulses with different durations on plated cells, cells grown on collagen layer and cells embedded in collagen gel (3D model) and analyzed gene electrotransfer efficiency. In order to analyze the effect of impaired mobility on gene electrotransfer efficiency, we applied electric pulses with different durations on plated cells, cells grown on collagen layer and cells embedded in collagen gel (3D model) and analyzed gene electrotransfer efficiency.

**Results:**

We obtained the highest transfection in plated cells, while transfection efficiency of embedded cells in 3D model was lowest, similarly as in in vivo. To further analyze DNA diffusion in 3D model, we applied DNA on top or injected it into 3D model and showed, that for the former gene electrotransfer efficiency was similarly as in in vivo. The experimental results are explained with theoretical analysis of DNA diffusion and electromobility.

**Conclusion:**

We show, empirically and theoretically that DNA has impaired electromobility and especially diffusion in collagen environment, where the latter crucially limits electrotransfection. Our model enables optimization of gene electrotransfer in in vitro conditions.

## Background

For DNA vaccination or gene therapy applications efficient delivery of plasmid DNA [1] or short RNA molecules [2] is crucial. Gene therapy is based on delivery of genes or alteration or removal of defective genes responsible for disease development [3]. The most efficient method used for gene therapy is viral transfection [4]. Although viral vectors have been very efficient, the safety of their use has been questioned [5–7]. Thus, there is a great interest in developing non-viral methods for gene delivery [8]. For the past 20 years a huge variety of non-viral gene therapy methods, including chemical and physical ones, have been developed to introduce DNA into the cell in vivo, but many of them are either toxic or have poor gene expression [8–11]. Almost four decades ago a physical method for delivery of molecules by use of electric pulses (electroporation) was described [12]. It is based on transient increase in the permeability of the cell plasma membrane caused by an externally applied electrical field. Electroporation is already successfully applied in different biomedical applications, including: electrofusion [13, 14]; electrochemotherapy [15, 16]; irreversible tissue ablation [17]; DNA vaccination [18, 19] and gene electrotransfer [20–22]. Today gene electrotransfer (GET) is widely used to introduce DNA into different cells [23, 24] and tissues [1, 25, 26] due to its safety and relatively easy application. GET is also used in a variety of clinical settings including cancer therapy, modulation of pathogenic immune responses, delivery of therapeutic proteins and drugs [27, 28]. Importantly, in the last decade DNA vaccination using electroporation became a very efficient approach in various settings, since it was demonstrated that electric pulses provide additional stimuli to the immune system [29]. DNA vaccination using electroporation has been successfully used for vaccination in different diseases, among others AIDS [30], various infectious diseases and very recently also for vaccination against COVID-19 [31].

Although the mechanisms of gene electrotransfer are not yet fully understood, it was shown that several steps are needed for successful transfection: (i) migration of DNA towards the cell; (ii) DNA insertion into the permeabilized cell membrane; (iii) DNA translocation across the membrane; (iv) migration of DNA towards the nucleus; (v) transfer of DNA across the nuclear envelope and finally; (vi) gene expression [22, 32–34].

Despite the fact that GET efficiency in vitro is quite high, efficient electrotransfer in in vivo conditions still presents a challenge. One of main causes of low in vivo electrotransfer efficiency is relatively low mobility of DNA in tissue compared to mobility in in vitro conditions. In different tissues, extensive network of extracellular matrix hinders DNA mobility to migrate towards the cell by reducing especially its diffusion and its electrophoretic mobility during electric pulse application [35–39].

Many parameters have been described, which may influence the efficiency of GET in vitro [32, 40–53] and in vivo [54–63]*.* Several studies have also shown that more efficient transfection can be achieved by using the combination of high-voltage (HV) short duration pulse, followed by a different number of low-voltage (LV) long-duration electric pulses [56, 64–67]. It was suggested that HV pulses are crucial for permeabilization of cell membrane, while LV pulses electrophoretically drag DNA to the cell. Also changing the polarity of the electric field during the electric pulse delivery was shown to increase gene electrotransfer as it allows interaction of DNA molecules with the larger surface area of the cell [68]. Moreover, different combinations of pulses were used in order to induce alteration of the nuclear envelope and to enhance gene electrotransfer efficiency [69]. Nevertheless, there is still a need for additional in vivo studies in order to overcome the problem of poor DNA migration towards the cell, which presents the first step needed for successful gene electrotransfer.

There is a great potential for 3D models in various fields of research in order to complement more traditional testing methods [70], to improve treatment planning [71], to validate protocols in order to forestall invasive surgical procedures [72] and to propose a reliable alternative to animal experiments [37]. Moreover, there are also diverse electroporation-based applications, exploiting 3D models either to study electropermeabilization [73], irreversible electroporation [74], electrochemotherapy [75] or gene electrotransfection [38, 76]. Successful gene electrotransfer was achieved only on cells located on the surface of 3D model, since these cells were in close contact with the added plasmid DNA solution. In addition, GET efficiency was strongly dependent on DNA mobility within tissue-rich in collagen [38].

A 3D model of Chinese hamster ovary cells (CHO cells) embedded in a 3D collagen matrix which we developed in [37] enables analysis of DNA electromobility and optimization of GET protocols. 3D in vitro models represents a valid biological tool allowing the analysis of various mechanisms with specifically defined parameters [76, 77].

This study aimed to analyze different parameters of GET in a 3D collagen model and to theoretically analyze DNA diffusion and electrophoretic mobility in relation to GET efficiency. Cells were grown: (i) in a monolayer, (ii) on top of a collagen layer or (iii) embedded into a 3D collagen gel. Different electroporation pulse parameters were investigated: duration, HV + LV combinations and pulse polarity. Moreover, two DNA protocol applications were used: DNA applied on top, or injected into the 3D model mimicking in vivo pDNA administration.

## Results

### Experimental results

To assess the effect of DNA mobility on gene electrotransfer efficiency, cells were grown as a standard in vitro monolayer culture, grown on top of a collagen layer, or embedded in a 3D collagen gel model [37]. DNA was applied on the top of the 3D gel.

**Fig. 1:**
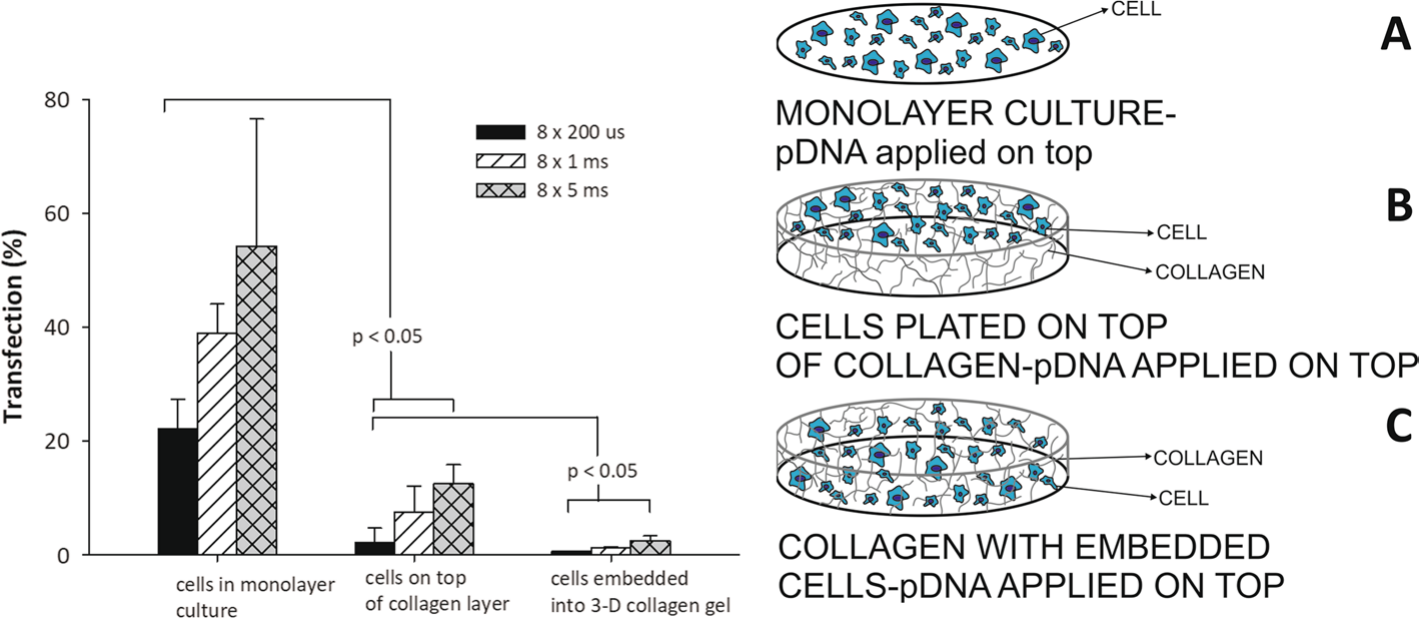
Gene electrotransfer efficiency (left) for different cell models. Cells were grown: (**A**-right) in a monolayer, (**B**-right) on top of collagen layer and (**C**-right) embedded into collagen gel (3D model). Eight pulses of different durations (200 µs, 1 ms or 5 ms), pulse repetition frequency of 1 Hz and *E *= 0.8 kV/cm were applied. pDNA concentration in electroporation media was 90 μg/ml. Values represent means (all tests were performed in triplicate) and error bars are determined from standard deviation. Results were considered as statistically different when *p* < 0.05

In Fig. 1-left the efficiency of gene electrotransfer for different pulse durations of plated cells (monolayer culture) (Fig. 1A-right), of cells grown on top of collagen layer (Fig. 1B-right) and of cells embedded in 3D collagen gel (Fig. 1C-right) are shown. We observed, that gene electrotransfer efficiency was always significantly higher when cells were plated as a monolayer culture compared to cells grown on top of the collagen gel. Also, more cells were successfully transfected when they were grown on top of collagen layer compared to cells embedded in 3D model. The highest electrotransfer efficiency was obtained when we applied 8 × 5 ms, *E* = 0.8 kV/cm pulses. Under this condition 54.2% of plated cells, 12.5% of cells grown on top of collagen layer and 2.5% of cells embedded in 3D model were transfected, while for shorter pulses transfection efficiency was significantly reduced.

**Fig. 2:**
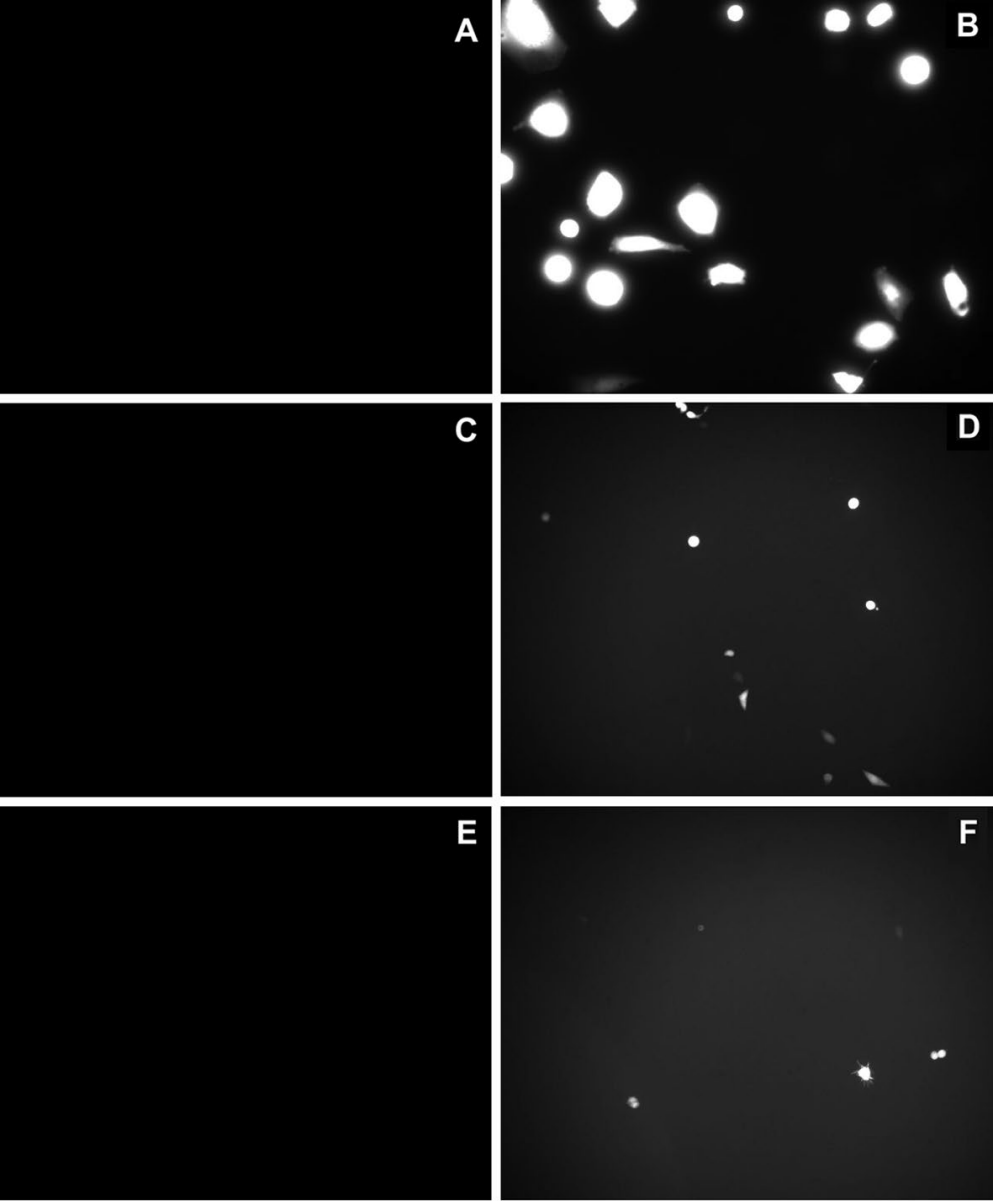
Representative fluorescent figures of different cell models. Cells that were expressing fluorescent GFP protein were defined as successfully transfected (successful gene electrotransfer was achieved). Cells were grown: **A** and **B** in a monolayer, **C** and **D** on top of collagen layer and **E** and **F** embedded into collagen gel (3D model). Concentration of added pDNA in electroporation medium was 90 µg/ml (**A**–**F**). Images of gel before pulses were applied (**A**, **C**, **E**), while in **B**, **D** and **F** eight pulses with 5 ms duration, pulse repetition frequency of 1 Hz and *E* = 0.8 kV/cm were applied. To visualize cells, × 20 (**A**, **B**) or × 10 (**C**–**F**) objective magnification was used

In Fig. 2, fluorescent images of cells, before and after pulse application are shown for all three models (cells in monolayer culture—Fig. 2A, B, cells grown on top of collagen layer—Fig. 2C, D and cells embedded in 3D collagen gel—Fig. 2E, F) for *E* = 0.8 kV/cm, 8 × 5 ms pulses with 1-Hz repetition frequency. Gene electrotransfer efficiency was higher when cells were plated as monolayer culture (Fig. 2B) compared to cells plated on top of the gel (Fig. 2D) or embedded inside the gel (Fig. 2F).

Several studies have demonstrated that applying LV pulses following the HV pulses enables more efficient electrotransfer in in vivo conditions [56, 64–67]. In order to determine if HV + LV protocol contribute to higher gene electrotransfer efficiency compared to HV in our 3D model similarly as in tissue, we further analyzed the effect of high-voltage (HV) and low-voltage (LV) pulses on gene electrotransfer efficiency. We used different combinations of HV and LV pulses, the parameters are presented in Table 1 (M&M). Furthermore, pulses with alternating polarities were used (Table 1) to evaluate the effect of such pulsing protocols on gene electrotransfer efficiency in a 3D model.

**Table 1:** Pulsing protocols for gene electrotransfer in 3D model

Protocol	Electric pulse parameters
HV 1	5 × 1 ms; 0.8 kV/cm; 1 Hz
HV 2	8 × 200 µs; 0.8 kV/cm; 1 Hz
LV 1	1 × 100 ms; 75 V/cm
LV 2	1 × 100 ms; 150 V/cm
Single polarity pulses—SP	8 × 1 ms; 0.8 kV/cm; 1 Hz
Orthogonal both polarities—OBP	8 × 1 ms; 0.8 kV/cm; 1 Hz

The time lag between HV and LV pulse was always 20 ms

**Fig. 3:**
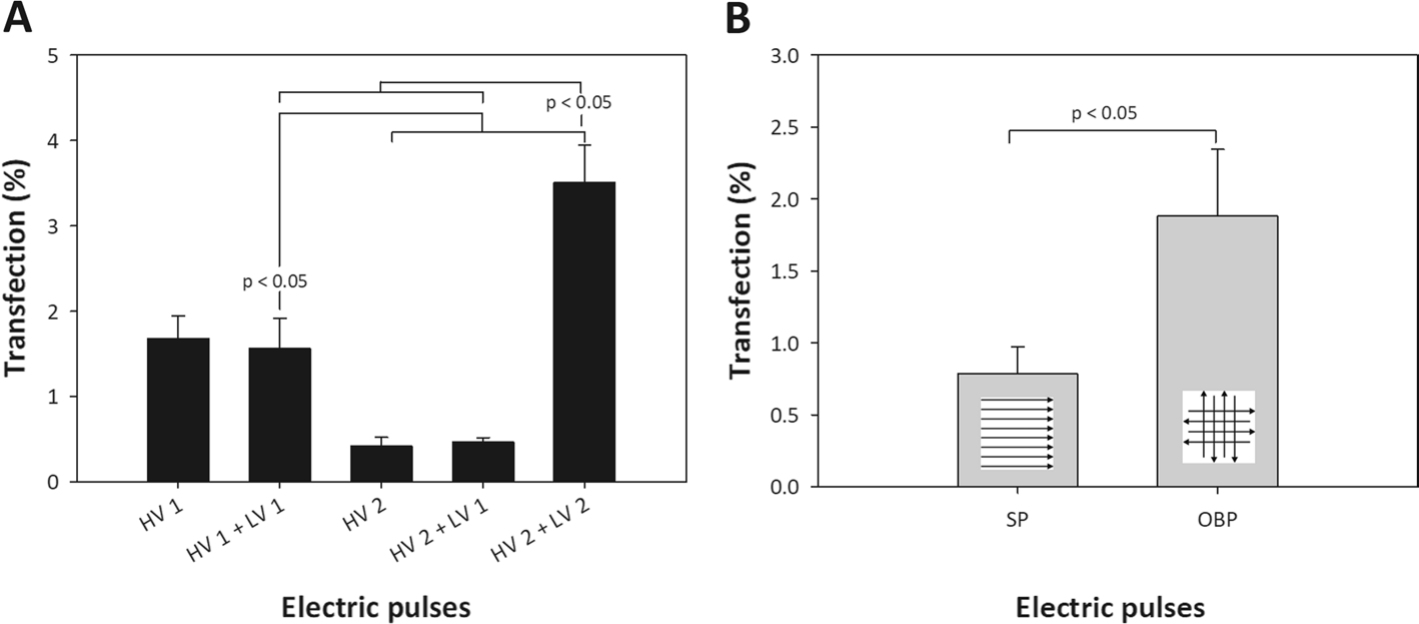
Effect of different pulsing protocols on gene electrotransfer efficiency (percentage of transfected cells) in a 3D collagen model with embedded CHO cells, pDNA was administrated on top of the gel. A Different combinations of high-voltage (HV) and low-voltage (LV) pulses were applied. The electric pulse parameters were as follows: HV 1 (5 × 1 ms; 0.8 kV/cm; 1 Hz), HV 2 (8 × 200 µs; 0.8 kV/cm; 1 Hz), LV 1 (1 × 100 ms; 75 V/cm) and LV 2 (1 × 100 ms; 150 V/cm). The time lag between HV and LV pulse was 20 ms. B 8 × 1 ms pulses (1 Hz) and *E* = 0.8 kV/cm with single polarity (SP) or orthogonal both polarities (OBP) were applied; cells were embedded in collagen gel (3D model). pDNA concentration in electroporation media was 90 μg/ml. Values represent means (all tests were performed in triplicate) and error bars are determined from standard deviation. Results were considered as statistically different when *p* < 0.05

In Fig. 3A, gene electrotransfer efficiency is presented for different combinations of HV and LV pulse protocols for cells embedded in collagen gel (3D model). When 5 × 1 ms pulses (HV 1), with *E* = 0.8 kV/cm were applied addition of LV1 pulse—1 × 100 ms; 75 V/cm (HV1 + LV1) did not contribute to gene electrotransfection efficiency. However, we obtained a significant increase (*p* < *0.05*) in transfection efficiency when HV2 pulses were combined with higher amplitude of LV pulse (HV 2 + LV 2), (8 × 200 µs; 0.8 kV/cm; 1 Hz + 1 × 100 ms; 150 V/cm), where approximately 3.5% of cells in 3D model were successfully transfected. Applying only LV pulse, no transfection was obtained (data not shown).

Furthermore, we analyzed if pulses with different polarities improve electrotransfection efficiency in a 3D collagen model. In Fig. 3B, percentage of transfection is presented for pulses with single or orthogonal both polarities for cells embedded in collagen gel. Higher gene transfer in 3D model was obtained, when pulses with different polarities were used (OBP) compared to single polarity pulses (SP) in agreement with in vitro study in standard in vitro monolayer cell culture [68]. For OBP pulsing protocol (8 × 1 ms, *E* = 0.8 kV/cm), 1.88% of cells in 3D model were successfully transfected.

**Fig. 4:**
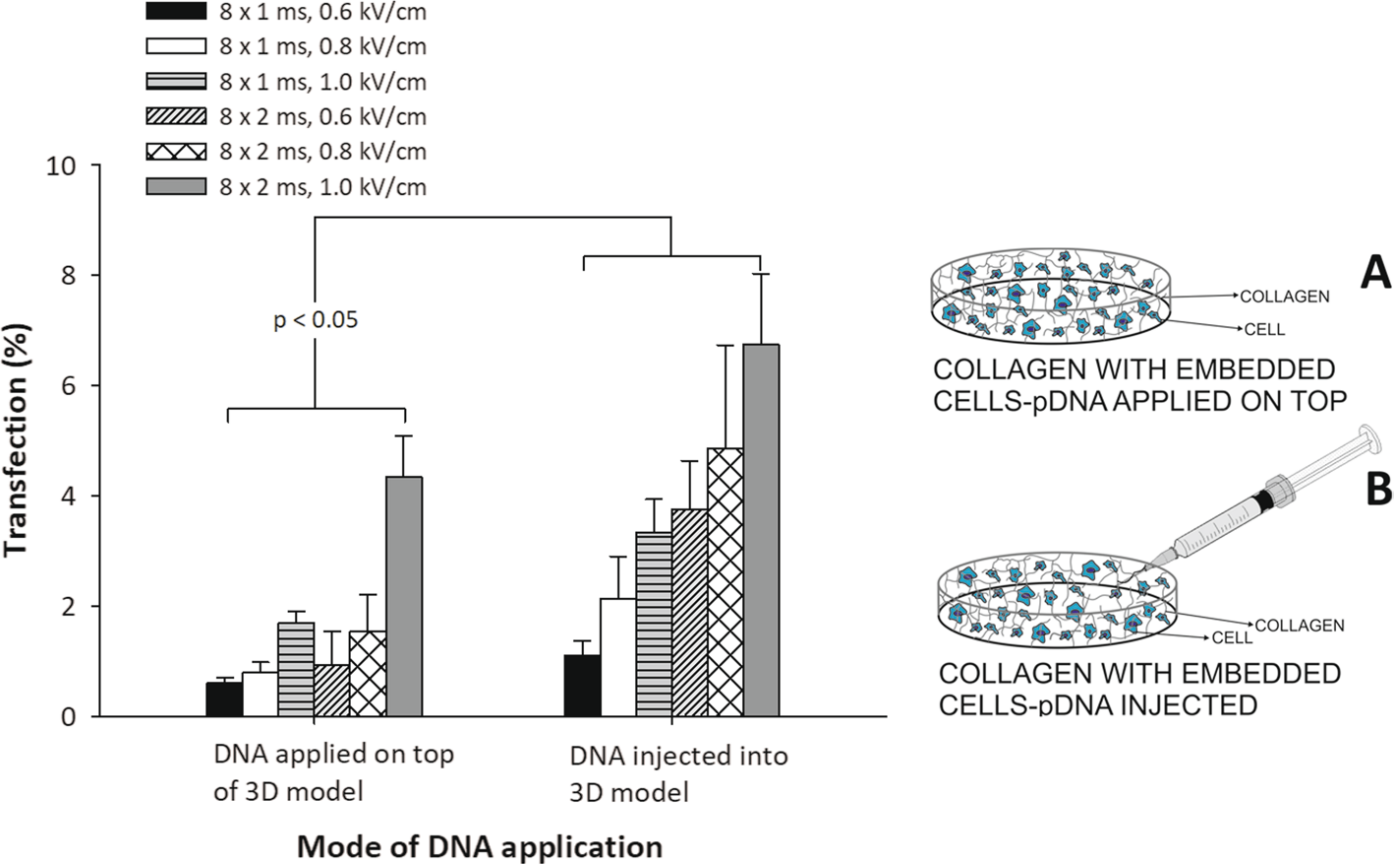
The effect of different modes of DNA administration on gene electrotransfer efficiency (left). Cells were embedded in a collagen gel and DNA was administered: on top of 3D model (**A**-right) or injected into the 3D model (**B**-right). The percentage of transfected cells is plotted for different electric pulses: 8 × 1 ms and 8 × 2 ms pulses of various electric field strength *E* (kV/cm). In all experiments, the same amount of pDNA was administered (18.2 μg). Values represent means (all tests were performed in triplicate) and error bars are determined from standard deviation. Results were considered as statistically different when *p* < 0.05

To further analyze how reduced mobility of pDNA inside a 3D collagen matrix affects gene electrotransfer efficiency, we compared gene electrotransfer efficiency for two different pDNA administration procedures: in the first, pDNA was administered on top of the 3D model, and in the second, injected into the 3D model (Fig. 4). In general, for both methods of pDNA administration the increase in gene electrotransfer efficiency was observed when longer pulses or pulses with higher E were applied. For pDNA injected into the 3D model, we consistently obtained statistically significant (*p* < 0.05) higher gene electrotransfer efficiency compared to pDNA applied on the top of a 3D model. The highest efficiency for both methods of pDNA application was obtained when we applied 8 × 2 ms pulses with the applied electric field *E* = 1.0 kV/cm. When pDNA was injected into 3D model 6.7% of cells were transfected, while for conditions where pDNA was applied on top of 3D model, the transfection efficiency was decreased to 4.3%.

### Theoretical analysis

The transport of ions and charged molecules is driven by diffusion and electric forces. Therefore, the flux of charged molecules (**J**) is described by Nernst–Planck equation:

1 $${\mathbf{J}} = - D\;\nabla c - D\,c\frac{e}{{k_{{\text{B}}} T}}\nabla \Psi,$$

where *D* is the diffusion constant of a specific molecule in a given medium, *e* the electric charge of a molecule, *c* the concentration distribution, *T* absolute temperature, *k*_B_ Boltzmann constant and Ψ electric potential. Both, the concentration gradient (diffusive part) as well as the potential gradient (electromobility part), contribute to the flux, where the latter dominates in case of very charged molecules and high electric fields. Here, we have to note that in an electrolytic solution even in the absence of current, the ions and charged molecules such as DNA do not diffuse independently.

#### Diffusion of pDNA inside collagen matrix—calculation of the concentrations distribution in a 3D gel

In this section, we will calculate movement of pDNA due to pure diffusion and we will thus neglect the electromobility part of the Nernst–Planck equation (Eq. 1). All DNA molecules are polyelectrolytes that have large negative charge therefore their concentration gradient will consequently lead also to the electric potential gradient. However, since in all physiological and culture media we have ionic solution, the ions will also be redistributed, therefore the second term can be neglected when the external electric field is zero. Therefore, the movement of pDNA before pulse application can be described by pure diffusion.

Due to our specific geometry where pDNA was applied on top of the gel (see Fig. 4A), the concentration distribution of pDNA before pulse application (*E* = 0) can be calculated with a diffusion equation in 1D:

2 $$c(z,t) = \frac{1}{{\sqrt {4\pi Dt} }}\int\limits_{{ - \infty }}^{\infty } {c_{0} } \left( {z{'}} \right)\;\exp \left[ { - \frac{{\left( {z - z{'}} \right)^{2} }}{{4Dt}}} \right]{\text{d}}z{'}\;.$$

**Fig. 5:**
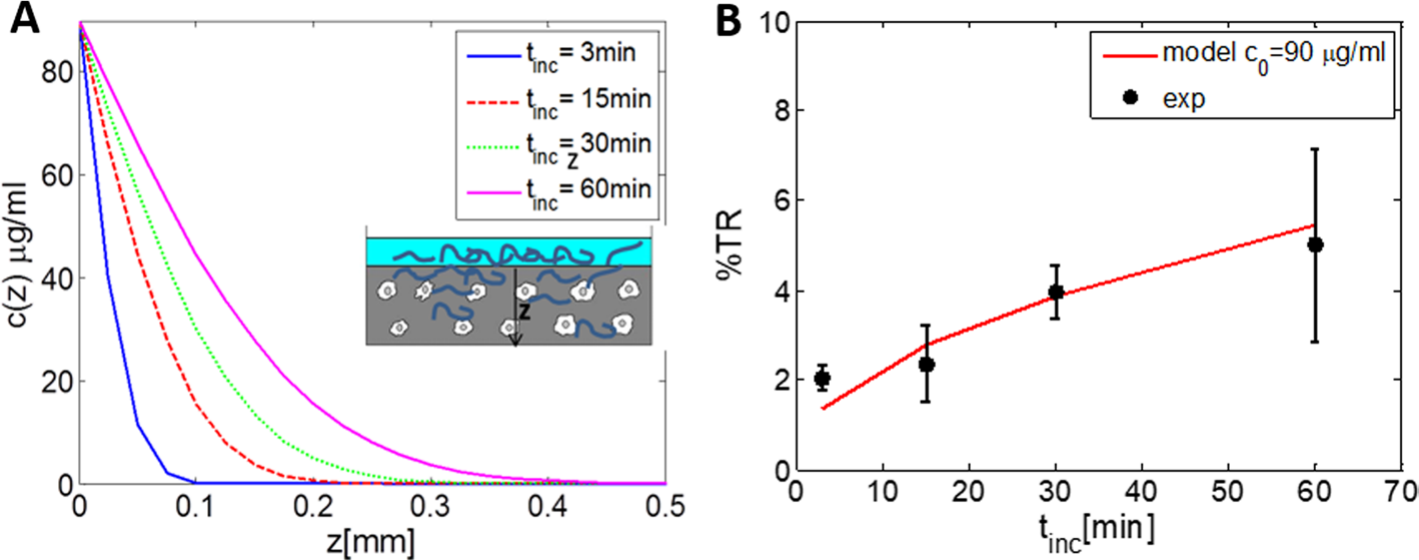
**A** Diffusion of pDNA into 3D collagen gel, where pDNA was applied on the top of the 3D gel. The distribution profile *c* (*z*, *t*_inc_) is calculated from Eq. (4), where z is the distance from the top of 3D gel and *t*_inc_ is the time of pDNA application on the top of the gel, the diffusion constant is: *D* = 3 × 10^–8^/cm^2^. **B** Comparison of the theoretically calculated % electrotransfection (%TR) based on the diffusion model of pDNA in 3D gel (Eqs. 4–6) and experimental values (exp) for different times *t*_inc_. Initial pDNA concentration applied on the top of the gel was *c*_0_ = 90 µg/ml, 8 × 5 ms electric pulses, *E* = 0.8 kV/cm were applied. Results are presented as a mean ± standard deviation

If we now consider our specific case where pDNA was applied on the top of the collagen gel (*z* = 0), a 1D diffusion equation in half space can be applied:

3 $$c(z,t) = \frac{1}{{\sqrt {4\pi Dt} }}\int\limits_{0}^{\infty } {c_{0} } \left( {z{'}} \right)\;\exp \left[ { - \frac{{\left( {z - z{'}} \right)^{2} }}{4Dt}} \right]{\text{d}}z{'}\; = \;\;c_{0} \,{\text{erfc}}(z/2\sqrt {Dt} )\;,\quad$$

where *c* (*z*, *t*) describes a time-dependent spatial concentration distribution of pDNA and *z* is the distance from the top of the gel to the given point inside the gel (see Fig. 5A). For estimation of the diffusion constant *D,* we have used measured diffusion coefficients from Zaharoff and Yuan 2004 [36] for 0.5–3% agarose gel. Since our 3D collagen gel with embedded cells is much less dense we have extrapolated curve from Fig. 5 [36] to lower gel percentages (0.35% w/w collagen) and obtained *D* = 3 × 10^−8^cm^2^/s, which we have used in our theoretical analysis of pDNA diffusion. In a more dense 3% gel *D* is reduced to ~ 0.01 × 10^−8^cm^2^/s, while in water media (culture media) *D* ~ *5* × 10^−8^cm^2^/s.

From Eq. (3), we can obtain the time-dependent concentration distribution for the given initial concentration distribution *c*_0_ (*z*, *t* = 0). For any given time of pDNA incubation (*t* = *t*_inc_), one can thus calculate the spatial concentration distribution of DNA *c* (z, *t*_inc_):

4 $$c(z,t_{{{\text{inc}}}} ) = c_{0} \;{\text{erfc}}(z/2\sqrt {Dt}_{{{\text{inc}}}} ).$$

Since electrotransfer efficiency depends on the local concentration *c* (*z,* *t*_inc_) of pDNA in the vicinity of a cell, it is important to relate pDNA concentration with the probability of transfection. In gene electrotransfer experiments, usually percentage of transfected cells is evaluated (%TR) and this parameter is directly dependent on the probability of transfection of a single cell—*P*_1,_ where for given *z* we obtain:

5 $$P_{1} (z) = K\; \times c\,(z,\,\;t_{{{\text{inc}}}} ).$$

Thus, percentage of transfected cell (%TR) is proportional to the integral of the concentration distribution for *z* = [0, *d* − thickness of a 3D gel] of all probabilities *P*_*i*_:

$$\% {\text{TR}} = K\;\int\limits_{0}^{d} {} c(z,t_{{{\text{inc}}}} ),$$

where constant *K* is proportional to the number of cells and other parameters that determine the final probability of transfection (e.g., pulse parameters). For our conditions the thickness of 3D gel is *d* = 0.95 mm. We have determined *K* based on our experimental results for plated CHO cells [32] where %TR approximately linearly increased with pDNA concentration up to 10 µg/ml. The next assumption which we used is that very high pDNA concentrations are toxic [78] and therefore also reduce transfection efficiency above *c* > 40 µg/ml.

From the above equations, one can calculate how pDNA diffuses inside and through the collagen matrix for the case where pDNA is administrated on the top of a 3D collagen. The diffusion equation Eq. (4) enables us to calculate the concentration distribution *c* (*z*, *t*_inc_) depending on the distance from the top of 3D gel (*z*) and on the time of incubation *t*_inc_*.* In Fig. 5A, we present calculated distribution of pDNA concentration—*c* (*z*) after diffusion for different times of pDNA incubation before the application of the electric pulses, where at *t* = 0 we added suspension with pDNA on the top of the gel. It can be seen, that in 1 h pDNA will penetrate few hundreds of micrometer inside the collagen gel. In order to reach 1 mm (bottom of the well), very long incubation time would have to be used.

**Fig. 6:**
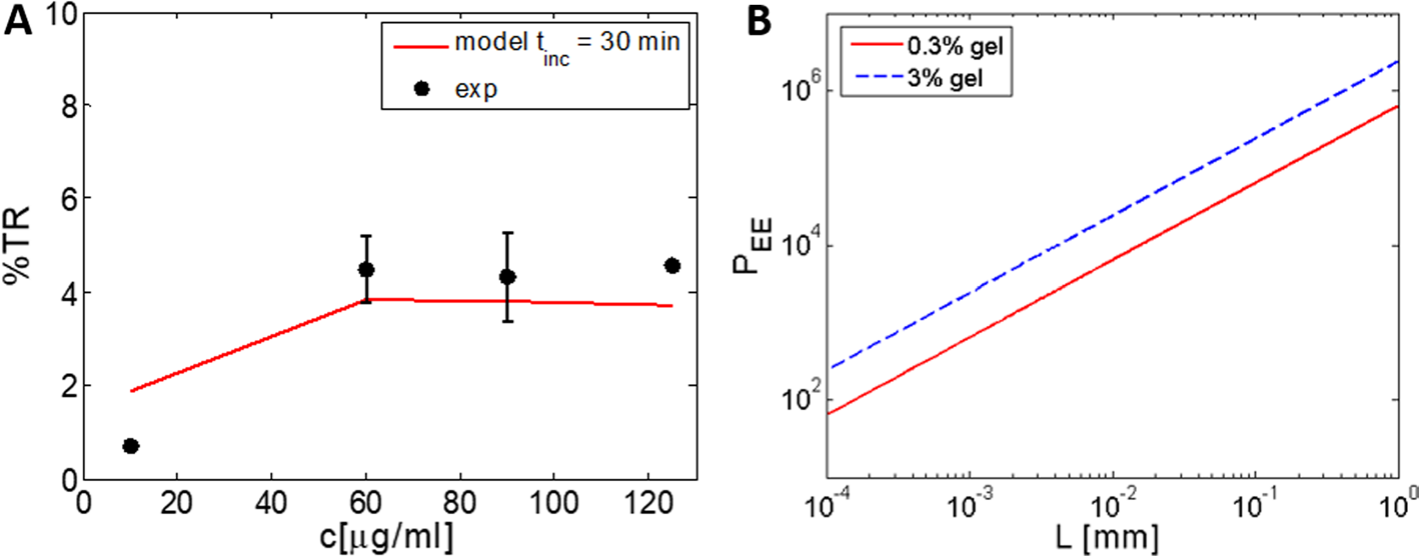
**A** Comparison of the theoretically calculated % electrotransfection (%TR) based on the diffusion model of pDNA in 3D gel (Eqs. 4–6) and experimental values for initial pDNA concentrations c_0_ and for following parameters: *t*_inc_ = 30 min, *D* = 3 × 10^–8^/cm^2^ and 8 × 5 ms electric pulses, *E* = 0.8 kV/cm. Experimental results (exp) are presented as a mean ± standard deviation. **B** Comparison of electrophoresis versus diffusion for a given transport distance *L*; a dimensionless parameter *P*_EE_ is defined as $P_{{{\text{EE}}}} = \mu \,E\,\; \times \,L/D\,$ where μ is electrophoretic mobility (see Sect. 3.2.3)

From the calculated pDNA distribution *c* (*z,* *t*_*inc*_), we can calculate the % electrotransfection (%TR) by integrating probability of transfection for all planes over *z* according to Eq. (6). In Figs. 5B and 6A, we show comparison of the experimental values with the theoretically calculated %electrotransfection based on the diffusion model (Eqs. 4–6) for different incubation times *t*_inc_ and different initial pDNA concentrations—*c*_0_ applied on the top of the gel. It can be seen that it is crucial to allow enough incubation time with pDNA before pulse application, in order to achieve efficient transfection and that above some maximal initial plasmid concentration %TR is not increased.

The theoretical model can approximately describe the experimental dependency on *t*_inc_ and *c*_0_. For a more dense gel, the same equations can be applied (Eq. 4), but the diffusion coefficient would be much lower, for example for a 3% gel ~ 300 × lower. In general the diffusion decreases exponentially with the gel concentration [36], therefore in dense tissue pDNA diffusion is very limited, in range of µm.

#### Quantifications of pDNA electrophoresis inside 3D collagen gel

Next, it is important to evaluate electrophoretic mobility of pDNA due to electric field (the second term in Nernst–Planck equation). Electrophoresis of DNA (electromobility) is a mechanism, which was shown to be important for the delivery of DNA molecules into cells by electric pulses [32, 34–36]. During pulse application, the electrophoretic driving force acts (*F*_E_) on the negatively charged DNA molecule and drags it toward the cathodic side of the cell membrane. It depends on the local electric field (*E*) and on the effective charge of a given molecule:

7 $$F_{{\text{E}}} = e_{{{\text{eff}}}} E,$$

where the effective charge depends on the ionic strength of the solution and length of the pDNA, *e*_eff_ = 0.066 per base pair × 4.7 kbp for our 4.7 kbp pDNA. DNA molecule moving in an aqueous solution under external electric field *E* reaches the steady-state velocities *v* practically immediately—in ~ 3 × 10^–11^ s [36], therefore during pulse application steady-state conditions can be assumed. Under steady-state condition frictional force *f* equals electrophoretic force (*F*_E_ = *f*), therefore electrophoretic mobility *μ* is:

8 $$\mu = e_{{{\text{eff}}}} /f\; = \frac{{e_{{{\text{eff}}}} }}{{6\pi \eta R_{{\text{g}}} }},\quad v = \mu E,$$

and depends on the friction drag *f* and the effective charge. For standard in vitro experiments in suspension or on plated cells, one can use the viscosity of water *η* as the buffer viscosity.

Clearly, in a 3D-gel matrix such as extracellular matrix, agarose or collagen gel the friction *f* and viscosity depend on the composition of collagen matrix. Furthermore, the above equations are valid for supercoiled pDNA for which we can assume globular geometry (Ogston sieving model). However, as described in different studies [35, 36] electrophoresis of pDNA inside 3D gels is a complex function of electric pulse parameters and density of the gels [79]. Thus, direct analytical calculation from Eq. (8) is not possible and measured electrophoretic mobilities *μ* from Zaharoff and Yuan [36] for 0.5–3% agarose gel were used to extrapolate their values [36] for our less dense (0.35% w/w) collagen gel (Table 2) and our pulsing parameters (8 × 200 μs, 8 × 1 ms, 8 × 5 m, 8 × 10 ms). From the estimated mobility, we can calculate the displacement *L*_E_ due to the electrophoretic displacement during the *N* pulses of total duration *t*_E_:

9 $$t_{{\text{E}}} = N \times t_{{\text{p}}} ,\quad L_{{\text{E}}} = v\;t_{{\text{E}}} \; = \mu \,E\,N\,t_{{\text{p}}},$$

from which we obtained that the maximal distances of pDNA movement *L*_E_ in our 0.3% gel for 8 × 5 ms are few tens of μm (Table 2). However, for more dense gel and tissue [80] the electrophoretic movements of DNA is severely reduced to around only ~ 1 μm. In Table 2, the electrophoretic displacement *L*_E_ due to electrophoresis in relation to the measured %TR and to factor *U*^2^ × *t*_E_ (proportional to electric energy of the pulses) are presented. Namely, one hypothesis is that the electric energy needed for DNA interaction with the membrane, is the crucial parameter for electrotransfection, since there exists an energy barrier between the negatively charged pDNA and the negatively charged cell membrane. In the most simplified case, we can thus assume that the electric energy of the pulses *W*_e_ equals the work of the electrophoretic force *A*_e_ = *F*_e_ × *L*_e_, which is proportional to the total length of pulses and square of the applied voltage *U*^2^ × *t*_E_ [34].

**Table 2:** The relation between the electrophoretic movement of pDNA (*L*_E_), measured %TR, electric pulse energy given in terms of *U*^2^ × *t*_E_ (also proportional to Joule heating *Q*_Joule_) and electromobilities ***μ*** (obtained from [36] for a less dense gel (0.35% w/w collagen) and more dense gel (3% collagen) for different length of the electric pulses: 8 × 200 μs, 8 × 1 ms, 8 × 5 ms, 8 × 10 ms; the applied electric field *E* = 0.8 kV/cm

Pulse parameters *E* = 0.8 kV/cm	Electromobility *μ* 0.35% gel (m^2^/Vs)	Electromobility *μ* 3% gel (m^2^/Vs)	*L*_E_ 0.35% collagen (μm)	*L*_E_ 3% collagen (μm)	*U*^2^ × *t*_E_ (V^2^s)	%TR (0.35% collagen)
8 × 200 μs	0.2 × 10^–8^	0.03 × 10^–8^	0.256	0.0384	40.96	0.65
8 × 1 ms	1 × 10^–8^	0.03 × 10^–8^	6. 4	0.192	204.8	1.55
8 × 5 ms	2.4 × 10^–8^	0.03 × 10^–8^	76.8	0.96	1024	3.58
8 × 10 ms	2.8 × 10^–8^	0.03 × 10^–8^	179.2	1.92	2048	2.16

From Table 2 it is clear that longer pulses increase electrophoresis and enable higher transfection (%TR). For very long pulses, electromobility reaches a plateau and for 8 × 10 ms, %TR is even decreased since very long pulses reduce cell viability. Namely, longer pulses increase the density of membrane defects, leading to higher DNA translocation into the cell. But if the number of membrane defects is too high, the cell fails to achieve biochemical balance leading to cell death [22, 40, 81–83]. Importantly, electric pulses produce Joule heating (*Q*_Joule_ = *I*^*2*^ × *R* × *N* × *t*_p_) that can result in significant increase in temperature (*Q*_Joule_ = *m* *c*_p_ ΔT). Furthermore, it was shown [84] that electric pulses can cause significant alterations in pH, where coulomb dosage is proportional to the electric current: *q* = *I* × *N* × *t*_p_ the length of the pulses. Joule heating and alterations in pH can lead to extensive cell death and plasmid damage resulting in reduced gene electrotransfection efficiency.

Similarly, as we have shown previously [34], we can estimate number of pDNA molecules *N*_*DNA*_ in the volume *V* = *S* × *L*_E_, which are available for the contact with the permeabilized part of the cell membrane from Eq. (8). The strength and length of the electric pulses *t*_E_ determine the distance *L*_*E*_ from which pDNA can access the cell and electric field strength determines the area of the membrane which is electropermeabilized *S:*

9 $$N_{{{\text{DNA}}}} = c(z,t) \times \,L_{{\text{E}}} \times \,S = c(z,t) \times \mu \,E\,t_{{\text{E}}} \; \times \,S.$$

From the above equation, it is clear, that the number of pDNA molecules available for contact with the membrane and consequently probability of electrotransfer linearly increases with the local pDNA concentration *c* (*z*, *t*) and electrophoretic displacement *L*_E_.

#### Electrophoresis versus diffusion

As already discussed, pDNA flux is described by Nernst–Planck equation where before pulse application diffusion dominates. During the pulses, strong electrophoretic force is acting on charged molecules such as DNA, however also diffusion is present. It is therefore important to evaluate pDNA transport due to electrophoresis versus diffusion on a given distance *L*. One can define a dimensionless parameter *P*_EE_ [36] that evaluates electrophoresis/diffusion ratio:

10 $$P_{{{\text{EE}}}} = vL/D = \mu \,E\,\; \times \,L/D,$$

where *v* is the electrophoretic velocity and *L* is the relevant transport distance. In Fig. 5B, *P*_EE_ is shown for 0.35% and 3% gel. For low-density gel, during electroporation electrophoresis dominates over diffusion on a 10-µm scale (cell diameter) for a factor of 5 × 10^3^, while for a more dense gel *P*_EE_ > 10^4^. For larger distances (1 mm) *P*_EE_ increases to > 10^6^.

## Discussion and conclusion

Gene electrotransfer is an established method to deliver genes both in vitro and in vivo. The main problem in gene electrotransfer of cells in vivo is still relatively low efficiency [22, 25, 26]. While in vitro, the DNA can easily reach cells and is therefore directly in contact with the cell membrane, which is one of the crucial steps in gene electrotransfer, in vivo, extracellular matrix hinders diffusion and electrophoresis of DNA consequently leading to relatively low transfection. Experiments in vitro and in vivo showed that for successful gene electrotransfer both electropermeabilization of the cell membrane and electrophoretic drag of plasmid DNA are needed. In vivo, mobility of pDNA is impaired, since tissue organization provides hindrance to the movement of DNA. Therefore, especially in tumors the highest transfection efficiency was ~ 5% [85]. In muscle cells, the transfection efficiency was higher due to specific properties of muscle cells [54].

Studying different parameters of gene electrotransfer in in vitro 3D gel—where especially mobility is drastically reduced—offers the possibility to study the mechanism and to enable optimization of the protocols for more efficient gene transfer in vivo.

In order to have more realistic in vivo model system, we used previously described 3D collagen model with embedded cells [37], which we have developed for analysis of the gene electrotransfer. Our 3D model was used to assess the impaired DNA mobility in a 3D multicellular environment with collagen gel representing extracellular matrix as a function of different electric pulse protocols. We used collagen gel density (0.35% w/w) which is in a range of collagen concentration of less dense tissues or tumors. For example, in the study of DNA mobility in tumors the authors experimentally determined the collagen concentrations in B16F10 tumors 0.252% w/w [35] and 2.44% w/w in 4T1 tumors [35]. In muscle tissue, the percentage of collagen is between 1–2%. Since collagen concentration directly influences the diffusion and electrophoretic mobility of DNA the presented 3D model can reproduce, hindered diffusion and electrophoresis of pDNA in tissues, which are two very important processes, involved for gene electrotransfer. However, in tumor tissue densely packed cells or other tissue structure can additionally hinder the mobility of DNA. The experiments were undertaken to gain understanding of impaired DNA mobility in a simple 3D in vitro model of a tissue-resembling environment (where cells are embedded into extracellular matrix) and to improve DNA delivery in vivo. We have to stress that our 3D collagen model is a simplified model of a fraction of tissue (e.g., tumor tissue), which is small enough to be homogenous, since cells are homogenously distributed inside gel. This is an approximation, since in vivo tissues have inhomogeneous structure and properties, where specific tissue structures and more dense cells and/or ECM can represent additional barrier that further affect mobility of DNA, as for example epidermis while in anisotropic muscle tissue DNA can diffuse along the fiber more easily then across the fibers [86].

In the first part of our study, we compared gene electrotransfer efficiency on: (i) plated CHO cells (standard monolayer culture); (ii) CHO cells grown on top of collagen layer (which represent the intermediate step between classical cell culture and in vivo model system) and (iii) CHO cells embedded in a 3D model. As we expected, gene electrotransfer efficiency was substantially higher when cells were plated as a monolayer culture, compared to cells grown on top of collagen layer or cells embedded in a 3D model for all pulsing protocols. Our experiments showed that maximum gene electrotransfer efficiency was obtained when pulses of longer duration were used. In this case, 54% of plated cells, 12% of cells grown on top of the collagen layer, and 2.5% of cells embedded 3D model, were successfully transfected. The difference in gene electrotransfer efficiency can be mostly explained by the fact that pDNA transport through the collagen matrix is relatively slow, especially when cells are embedded in the 3D model. Our results of %TR in 3D collagen in vitro model are comparable to the results of in vivo experiments, where similar gene electrotransfer (around 2%) was obtained [87].

Since it was shown by many in vivo studies [56, 64–67, 88] that short high-voltage (HV) microsecond pulses in combination with long low-voltage (LV) millisecond pulses contribute to higher gene electrotransfer efficiency, we analyzed in the second part of our study the influence of different combinations of HV and LV pulses on gene electrotransfer efficiency in 3D model. We obtained higher gene electrotransfer efficiency when using HV2 (8 × 200 µs; 0.8 kV/cm) pulse in combination with higher low-voltage pulse LV 2 (1 × 100 ms; 150 V/cm) pulse, compared to using only HV 2 pulse or lower LV 1 pulse. Our results are in agreement with previous in vivo [88] and in vitro studies [32, 89]. They showed that if longer pulse, with lower amplitude (LV pulses) is applied after shorter pulses with high amplitude (HV pulses) gene electrotransfer efficiency is increased. Namely, HV pulses permeabilize the membrane, while LV pulse is crucial for formation of a contact between pDNA and cell membrane [32, 35]. In addition, longer pulses enable increase in electromobility as shown in [35]. Moreover, as stressed in previous papers [35, 36] the advantage of electrophoretic drag of DNA over diffusion increases with both transport distance and gel concentration (Fig. 5B). A 3D gel can be considered as a distribution of larger voids connected by narrower passages or pores (*R*_*p*_), where their size depends on gel density: *R*_p_* ≅ 118* × *A*^*−*0.74^ [35, 79]*,* where A is concentration of the gel. For pDNA of *R*_g_ around 100 nm, it means that in less dense tissue (e.g., B16 tumors) where the pores size *R*_*p*_ in gel is around 270 nm the DNA can move relatively easy (Zimm model) [79]. When the radius of a molecule is comparable to the mean pore size (for 3% collagen 50 nm) or larger (*R*_g_ > *R*_p_*/2*), transport is significantly hindered by frictional and steric interactions between the pDNA molecule and the pores [35] and the movement can be described by reptation theory [36, 79]. Electrophoresis elongates pDNA in the direction of field and movements, and decreases its diameter in the perpendicular direction and therefore decreases entropy. The narrow passages function as entropic barriers to the transport of pDNA, thus application of longer pulses or HV-LV pulses could reduce the height of the entropic barrier.

However, in dense tissues or tumors (3% collagen) even with long pulses (e.g., 8 × 5 ms, *E* = 0.8 kV/cm) the electrophoresis during the pulses is very small ~ 1 μm (see Table 2)—therefore, we suggest that electrophoretic pulses are more crucial for formation of a contact between pDNA in vicinity with the membrane as we explain in our paper [34], but cannot be used to»drag« pDNA through extracellular matrix. This could also explain experimental data where in some studies HV-LV pulses could increase transfection efficiency in muscle tissue [64, 65], while in tumor tissue this effect was less pronounced. Also Heller et al*.* showed very efficient electrotransfection for only HV pulses [90].

In the second part of our study, we analyzed the influence of changing the electric field orientation on gene electrotransfer efficiency in 3D model. It was already suggested, that by changing the polarity of the pulses the membrane area that is competent for DNA entry into the cell increases. Our results in 3D model were consistent with previous results [41, 68] where gene transfection is increased when the electric field orientation between electrical pulses is changed (OBP protocol) compared to single polarity pulses, however the electrotransfer efficiency was low.

Furthermore, in vivo pDNA is usually delivered to the target cells by means of a local injection [55, 64–66] and consequently only cells in vicinity of injected site are in close contact with high concentrations of pDNA. Therefore, we next analyzed pDNA mobility in a 3D model by applying pDNA on top or injected it into the 3D model. We observed that the latter way of application showed higher transfection efficiency compared to the former one. The highest gene electrotransfer efficiency was obtained for both ways of pDNA application, when 8 × 2 ms pulses with *E* = 1.0 kV/cm was used. At those conditions, the highest transfection obtained was around 6.7% when pDNA was injected into 3D model, compared to ~ 4.3%, when pDNA was applied on the top of the 3D model. We also observed that more cells were successfully transfected near the injection site (data not shown).

In parallel, we present theoretical quantification of pDNA diffusion in collagen matrix that shows good agreement with the experimental results (see Figs. 5, 6B). We demonstrate that in a 3D gel model it is very important to allow long incubation time after application/injection of pDNA before pulse application allowing diffusion in larger area. In in vitro 3D model pDNA mobility due to diffusion is in range of few hundreds μm), while in real tissue that has more dense collagen structure as shown by [35] the diffusion is almost negligible (< 1 μm). Therefore, hindered pDNA mobility in ECM is one of main obstacles for efficient GET in vivo, since mobility in ECM is decreased for a factor of 100 or more compared to water, which extremely limits pDNA redistribution after injection and decreases number of DNA molecules in contact with the cell and leading to low transfection efficiency.

Thus, our results show that in dense extracellular matrix (e.g., in tumor tissue, skin, muscle), it is crucial to inject pDNA in several sites, thus enabling coverage of larger area of tissue with sufficient local pDNA concentration which will enable efficient gene electrotransfer [91]. Also, using low-voltage pulses after HV or orthogonal—both polarity pulses (see Fig. 3) can increase transfection efficiency in agreement with other studies [68, 92]. One strategy that was shown to improve electrotransfection efficiency in vivo in muscle [57, 93] and in tumors [94] is also to administer enzymes such as collagenase or hyaluronidase that disrupt extracellular matrix and enable better DNA mobility, however this strategy is potentially problematic in tumors due to potential dissemination of cancer cells. In addition, also other factors impair electrotransfer efficiency such as irreversible damage of cells due to irreversible electroporation (IRE) [55, 71], thermal damage [95, 96] due to Joule heating and pH changes [97, 98]. In addition, these processes can also damage DNA resulting in pure transfection. From the dependence of the electrophoretic force, Joule heating and coulomb dosage (pH alterations) on *E* it follows that it is most optimal to increase the length of the pulses to the point where viability is still preserved, while the voltage should be moderate and is usually close to the threshold for electropermeabilization [83, 84]. Indeed, in vivo studies [85, 88] and numerical modeling [95] have shown that more homogenous electric field distribution and optimizing the pulses in order to limit IRE can improve transfection. As we show here, in addition to optimization of the electric pulses, multiple injections of pDNA at different sites and application of enzyme to disrupt ECM can aid in better in vivo electrotransfection efficiency.

## Conclusions

To conclude, we show that our 3D collagen model resembles the in vivo situation more closely than the conventional 2D cell cultures and that the efficiency of gene electrotransfer and mobility of DNA in 3D model resembles the efficiency in in vivo environment. With theoretical analysis of pDNA diffusion and electrophoresis in 3D gel we demonstrate, that limited diffusion and electrophoresis of pDNA in ECM is one of the main limiting factors for GET efficiency and that in dense extracellular matrix of tissues it is crucial to inject pDNA in several sites. Thus, our 3D model provides an intermediate between in vitro and in vivo conditions to optimize the protocols for GET and to study mechanisms of gene electrotransfer for biomedical applications.

## Materials and methods

### Cell culture

For the experiment, Chinese hamster ovary cells (CHO-K1) were used (European Collection of Cell Cultures, Salisbury, UK). Cells were: (A) grown as a monolayer culture in 24-multiwell plate; (B) grown on top of collagen gel layer and (C) embedded in collagen gel (3D model), where DNA was applied on top or injected into 3D model (Figs. 1, 4). For cells, culture medium F-12 HAM (Dulbecco’s modification of EMEM) supplemented with 10% fetal bovine serum and 0.15 mg/ml L-glutamine (Sigma-Aldrich, St. Louis, MO, USA) was used.

#### Preparation of cells grown as a monolayer culture

CHO-K1 cells were plated as a monolayer culture (Fig. 1A-right) in Ham’s tissue culture medium in 24-multiwell plate in cell density of *ρ* = 5 × 10^4^ cells/ml (5 × 10^4^ cells/well). The plate was stored for 24 h at *37 °C* in a humidified 5% CO_2_ atmosphere in the incubator (Kambič, Slovenia).

#### Preparation of cells grown on top of collagen gel layer

Type I collagen from rat tail was obtained from Sigma-Aldrich Chemie GmbH (Deisenhofen, Germany) as a powder and mixed with diluted acetic acid (28.5 ml glacial acetic acid/liter) to achieve collagen solution concentration 4.0 mg/ml and stored at *4 °C.* After 24 h 1 × PBS, pH = 7.4 was added to collagen solution, in the ratio of 1:8. pH of mixture was adjusted to 7.2–7.6 with *0.1 M* NaOH. To prevent gelation, temperature of mixture was maintained at 2–8 °C. 200 μl of collagen was pipetted into each space of 24-multiwell plate and stored for 1 h at *37 °C* *i*n a humidified 5% CO_2_ atmosphere in the incubator. Collagen polymerized and formed a gel layer.

After 1 h incubation of collagen layer at *37 °C*, CHO-K1 cells were added on top of collagen layer as a monolayer culture (Fig. 1B-right) in Ham’s tissue culture medium in cell density of *ρ* = 5 × 10^4^ cells/ml (5 × 10^4^ cells/well). The plate was placed back into the incubator (*37 °C*, 5% CO_2_) for 24 h.

#### Preparation of collagen gel with embedded cells (3D model)

Collagen solution was prepared as described above. After 24-h incubation of collagen solution at *4 °C*, collagen mixture was prepared as already described before [37]. Briefly, 2.3 parts of chilled collagen solution was mixed with 0.5 part of Ham tissue culture medium for mammalian cells and 0.5 part of 1 × PBS, pH = 7.4. CHO-K1 were prepared as a cell suspension and cell pellet was re-suspended with liquid collagen solution to a cell density of *ρ* = 5.6 × 10^5^ cells/ml. 180 μl of collagen with cells (1.008 × 10^5^ cells/well) was pipetted into each space of multiwell dish and stored for 1 h at *37 °C* in a humidified 5% CO_2_ atmosphere in the incubator. After raising the temperature to *37 °C, *collagen polymerized and formed a gel with embedded cells inside (3D model) (Fig. 1C). Ham’s tissue culture medium was then gently added and cells were stored for 24 h at *37 °C* in a humidified 5% CO_2_ atmosphere.

### Plasmid DNA

Plasmid pEGFP-N1 (Clontech Laboratories Inc., Mountain View, CA, USA) encoding green fluorescent protein (GFP) was amplified in Top10 strain of *Escherichia* *coli* and isolated with HiSpeed Plasmid Maxi Kit (Qiagen, Hilden, Germany). Plasmid DNA (pDNA) concentration was spectrophotometrically determined at 260 nm and confirmed by gel electrophoresis.

### Gene electrotransfer

Our study was divided into three sets of experiments. In the first part, gene electrotransfer was performed on plated cells, on cells grown on top of collagen layer and on cells embedded in 3D model (to show how DNA mobility—which was lowest in 3D model—affects gene electrotransfer efficiency) (Fig. 1A–C, right). In the second part, we analyzed gene electrotransfer efficiency in 3D model by using different pulsing protocols—combinations of high-voltage and low-voltage pulses, single-polarity pulses and orthogonal both polarities pulses (to show, that also in our 3D model different pulsing protocols are affecting gene electrotransfer efficiency) and in the third part gene electrotransfer was performed on cells embedded in 3D model, where DNA was administered on top or injected into the 3D model (to show, how injected DNA can come closer to the cells and by that gene electrotransfer efficiency could be increased) (Fig. 4A, B-right).

Electroporation was performed on a 24-h-old cell culture with standard electroporation media (pH 7.4, 10 mM NaH_2_PO_4_/Na_2_HPO_4_, 1 mM MgCl_2_ and 250 mM sucrose). On the day of the experiment culture medium was removed and cells were incubated with 200 µl of electroporation media with pDNA that codes for GFP for 30 min at a room temperature (22 °C). Plasmid DNA concentration in electroporation media was 90 μg/ml.

**Fig. 7:**
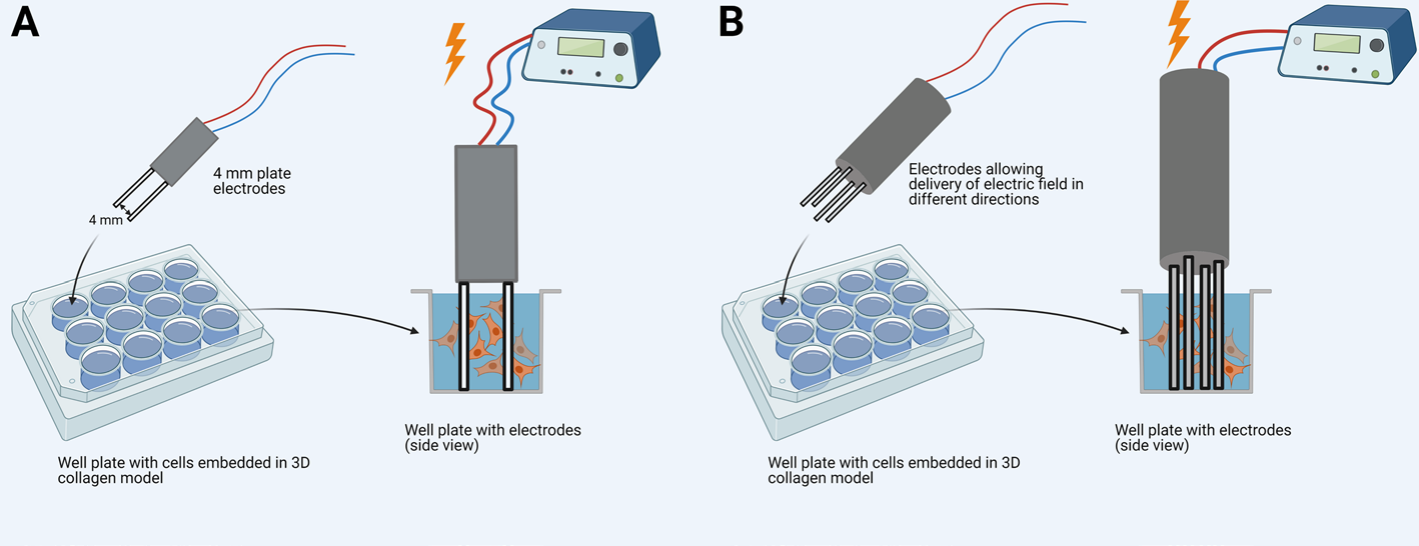
Experimental setup where different electrodes and their location are presented: **A** 4-mm plate electrodes were used for experiments, where pulse duration and different combinations of HV and LV pulses were studied; **B** electrodes allowing delivery of electric field in different directions were used, where the effect of pulse polarity was studied

In the first and third part of the experiment, a Jouan GHT 1287 pulse electroporator (Jouan, St. Herblain, France) was used; for pulse shape monitoring, a Wave surfer™ 422 (Le croy, Chestnut Ridge, New York, USA) was used. The distance between a pair of two plate stainless steel parallel electrodes was *d* = 4 mm (see Fig. 7).

In the second part of the experiment, a Cliniporator™ (IGEA s.r.l., Carpi, Modena, Italy) pulse generator was used. This enabled different combinations of high- (HV) and low-voltage (LV) pulses. The distance between a pair of two plate stainless steel parallel electrodes was *d* = 4 mm. For analyzing gene electrotransfer efficiency by changing pulse polarity a high-voltage prototype generator (EP-GMS 7.1) was used [68], which allowed application of relatively homogeneous electric field in different directions. An oscilloscope Wave surfer™ 422 (Le croy, Chestnut Ridge, New York, USA) monitored pulse shape. Especially designed electrodes allowing delivery of electric field in different directions and at the same time providing relatively homogeneous electric field distribution were used (see Fig. 7) [68]. No electric pulses were applied to cells in a control sample.

In the first part of the experiment, electroporation media with pDNA was applied on top of plated cells, cells grown on top of collagen layer and cells embedded in 3D model. A train of eight square wave pulses of different pulse durations: 200 μs, 1 ms and 5 ms were used to deliver pDNA into the cells. Electric field strength was 0.8 kV/cm, with repetition frequency 1 Hz for all pulsing protocols.

In the second part, electroporation media with pDNA was applied on top of cells embedded in 3D model. Different types of pulsing protocols were used to deliver pDNA into the cells as shown in Table 1. Also different incubation times and pDNA concentrations were tested.

In the third part, two ways of pDNA administration were studied in 3D model: (i) electroporation media with pDNA applied on top of 3D model or (ii) electroporation media with pDNA injected into the 3D model (see Fig. 4A, B). Electric pulses of two different pulse durations were used: 8 × 1 ms and 8 × 2 ms to deliver pDNA into the cells. Electric field strengths used were 0.6 kV/cm, 0.8 kV/cm and 1.0 kV/cm, with repetition frequency 1 Hz for all pulsing protocols.

After exposing cells to electric pulses, 70 µl of fetal calf serum was added (35% of sample volume) to preserve cell viability. Cells were then incubated for 15 min at 37 °C to allow cell membrane resealing and then grown for 24 h in cell culture medium at 37 °C in a humidified 5% CO_2_ atmosphere in the incubator.

Cells expressing fluorescent GFP protein were defined as successfully transfected (successful gene electrotransfer was achieved). Gene electrotransfer efficiency was determined by fluorescent microscopy (Zeiss 200, Axiovert, ZR Germany) with excitation light at 445 nm generated with a monochromator system (PolyChrome IV, Visitron, Germany) and emission was detected at 488 nm. The images were recorded using imaging system (MetaMorph imaging system, Visitron, Germany). At least ten fluorescence images were acquired in the area between the electrodes at 10 × objective magnification per each parameter. The cells were counted manually and gene electrotransfer efficiency was determined by the ratio between the number of green fluorescent cells (successfully transfected) and the total number of cells.

Total number of cells was difficult to determine from phase-contrast image in 3D model. For this reason, we first determined at which pulsing parameters the entire cell population was permeabilized to PI (8 × 5 ms pulses, *E* = 1.2 kV/cm, repetition frequency of 1 Hz). Therefore, after 5-min incubation with PI samples were exposed to electric pulses to permeabilize whole cell population using Jouan GHT 1287 electroporator. At least ten fluorescence images were acquired in the area between the electrodes at 10 × objective magnification per each parameter. The cells were counted manually.

### Statistical analysis

Experiments were repeated three or more times, on different days to prove repeatability and results are presented as mean values ± standard deviation. Results were evaluated using an unpaired *t*-test analysis (SigmaPlot 11.0, Systat Software, Richmond, CA, USA) and were considered as statistically different at *p* < 0.05.

## Data Availability

The datasets used and/or analyzed during the current study are available from the corresponding author on reasonable request.
